# Host-Specific Effects of Arbuscular Mycorrhizal Fungi on Two *Caragana* Species in Desert Grassland

**DOI:** 10.3390/jof7121077

**Published:** 2021-12-15

**Authors:** Xin Guo, Zhen Wang, Jing Zhang, Ping Wang, Yaoming Li, Baoming Ji

**Affiliations:** 1 School of Grassland Science, Beijing Forestry University, Beijing 100083, China; xinguo@bjfu.edu.cn (X.G.); zhangjing_2019@bjfu.edu.cn (J.Z.); 2 Grassland Research Institute, Chinese Academy of Agricultural Sciences, Hohhot 010010, China; wangzhen0318@126.com; 3 Command Center for Integrated Natural Resource Survey, China Geological Survey, Beijing 100055, China; wangping01@mail.cgs.gov.cn

**Keywords:** arbuscular mycorrhizal fungi, inoculum source, home advantage, *Caragana*, desert grassland

## Abstract

Arbuscular mycorrhizal fungi (AMF), which form symbioses with most land plants, could benefit their hosts and potentially play important roles in revegetation of degraded lands. However, their application in revegetation of desert grasslands still faces challenges and uncertainties due to the unclear specificity of AMF-plant interactions. Here, *Caragana korshinskii* and *Caragana microphylla* were inoculated with either conspecific (home) or heterospecific (away) AM fungal communities from the rhizosphere of three common plant species (*C. korshinskii*, *C. microphylla* and *Hedysarum laeve*) in Kubuqi Desert, China. AMF communities of the inocula and their home and away effects on growth and nutrition status of two *Caragana* species were examined. Results showed that AMF communities of the three inocula from *C. korshinskii*, *H. laeve* and *C. microphylla* were significantly different, and were characterized by high abundance of *Diversispora*, *Archaeospora*, and *Glomus*, respectively. The shoot biomass, photosynthetic rate, foliar N and P contents of *C. korshinskii* only significantly increased under home AMF inoculation by 167.10%, 73.55%, 9.24%, and 23.87%, respectively. However, no significant effects of AMF on *C. microphylla* growth were found, regardless of home or away AMF. Positive correlations between *C. korshinskii* biomass and the abundance of AMF genus *Diversispora* were found. Our study showed strong home advantage of using native AMF community to enhance *C. korshinskii* growth in the desert and presented a potentially efficient way to use native AMF in restoration practices.

## 1. Introduction

Arbuscular mycorrhizal fungi (AMF) provide benefits to their host plants [1,2] and show functional diversity assumed to have implications for revegetation of degraded lands [3,4,5]. By forming biotrophic symbiosis with plants, AMF could enhance plant uptake of relatively immobile nutrients, particularly phosphorus, and several micronutrients [6,7,8]. Besides, AMF could also provide a large number of other benefits to host plants such as maintaining and ameliorating soil structure, pathogen and herbivory protection, alleviation of drought and salinity stress [9,10,11,12,13].

However, the benefit of AMF in terms of increased plant growth is not always evident [14,15]. Neutral or negative effects of AMF on plant growth in both greenhouse [16,17] and field experiments [18] were reported. Furthermore, the effects of AMF on plant growth seemed to be host specific [19]. The AMF-plant symbiosis is not formed stochastically, but depends on their functional traits [20,21]. Both plant hosts and AMF have been shown to preferentially allocate resources to higher quality partners [22]. Consequently, due to the simultaneous processes of coevolution and bi-direction selection, the plant-AMF usually achieved good match for each other. In addition, the behavior of AMF was also dramatically affected by local soil properties, such as soil texture and structure, organic matter, and pH [23]. Thus, theoretically, the native AMF should benefit the local plant better than that from foreign habitats, which has been reported previously [4]. For example, native AMF enhanced the growth of *Sorghastrum nutans* more than fungi from an alternate system in the greenhouse [24]. However, the knowledge on the host specific benefits provided by AMF to plants obtained from greenhouses should be further tested in field studies.

Desertification is one of the most serious global environment problems, especially in China [25]. Specifically, 2.62 million km^2^ of the drylands, accounting for 27.33% of the country’s territorial area, is desertified and caused serious ecological problems [26]. In such desert land, characterized by low plant cover and soil fertility, the below-ground microorganisms were severely disturbed resulting in a relatively low level of beneficial soil microbes, such as AMF [27,28]. Usually, in northern China, most shrubs in arid regions are prevalently colonized by AMF and the symbiosis is critical to their survival and regrowth [29,30,31]. With the loss of AMF after desertification, the growth of the shrubs might be dampened [32,33]. *Caragana korshinskii* and *Caragana microphylla* are common brushes of the desert in northern China and are frequently used for revegetation due to its well adaptation to water and nutrient deficit [34,35]. Furthermore, they are extensively colonized by AMF and host specific interactions between *C. microphylla* and AMF was detected in desertified grasslands in northern China [36], indicating the potential importance of AMF in benefiting their growth. However, the effects of AMF on revegetation of *C. korshinskii* and *C. microphylla* are still not clear in practice. Thus, field experimental tests of the effectiveness of AMF on plant growth in desertified area and their proper applications are an urgent demand [3,4,5].

Here we investigate the inoculation effect of home (i.e., conspecific AMF inoculation) and away (i.e., heterospecific AMF inoculation) AMF on the growth of *C. korshinskii* and *C. microphylla* in Kubuqi Desert continuously for three years. The Kubuqi Desert is the seventh largest desert and is a major dust source in China. The home and away AMF inocula were obtained from the roots and soils of three host species (*C. korshinskii*, *C. microphylla* and *Hedysarum laeve*) in the desert. We hypothesized that: (1) AMF inoculation could enhance the plant performance in the desert; (2) AMF inocula from the rhizosphere of different host species differed in their ability to promote plant growth, and specifically, plants would grow better under home AMF relative to away AMF.

## 2. Materials and Methods

### 2.1. Study Site

The study site was located in the eastern part of the Kubuqi Desert in Zhungeer Banner in the north of the Erdos Plateau in Inner Mongolia, China (40°04′47.13″ N, 110°46′34.83″ E). The mean annual temperature here is 6.2–8.7 °C and annual precipitation is 420 mm with more than 60% of the precipitation occurring from July to September. The soil texture is arenosol and contained 1.0 g kg^−1^ soil organic carbon. The total N and available P were 0.09 g kg^−1^ and 4.36 mg kg^−1^ respectively. The site was previously dominated by natural occurring *Artemisia Ordosicaonce* but with a very low plant coverage (<10%).

### 2.2. Trap Culture of AMF

In June 2014, 10 individuals of *C. korshinskii*, *H. laeve* and *C. microphylla* with similar growth conditions were selected at the study site. Samples containing root and rhizosphere soils of each individual were collected from the top 0–15 cm using soil auger with 5-cm diameter and were mixed for each plant species. AMF spore density in these samples were measured by wet-sieving (paired sieves: 750 and 38 μm) and sucrose-gradient centrifugation [27]. The spore density was 133 ± 26, 123 ± 38, and 98 ± 47 spores per 100 g soil for *C. korshinskii*, *H. laeve* and *C. microphylla*, respectively (Figure S1). Desert soil samples nearby were also collected at the same time and steam-sterilized (121 °C, 1 h, twice) to serve as growth substrate for following trap culture of AMF. The collected root and rhizosphere soil samples of the three shrubs containing AMF community were mixed with sterilized growth substrate in a 1:2 ratio (*v/v*) in 20 sterilized plastic pots (20 cm diameter ×15 cm depth), respectively. Then, 20 seeds of maize (sterilized with 75% alcohol) were sown on the surface of each plastic pots. To equalize microbial communities of the trap culture soils except AMF, soil sievates containing microbes except AMF from the three shrubs were added to the pots. The field soil sievates were obtained by blending the collected root and soil samples and water in a 1:2 ratio and passing the slurry through a 38 μm sieve. The relatively large AM fungal spores and hyphae were trapped on the sieve, while smaller organisms passed through [37]. All trap culture pots were amended with 150 mL non-sterilized soil sievates, with 50 mL from each of the three species. Supplied with tap water when needed, maize was grown for 14 weeks at a temperature of 25 °C in a greenhouse to obtain enough AMF spores. Then, the above ground parts of the maize were removed, and all contents underground were harvested separately as inocula for the following experiment. Maize roots were removed from the soil, cut into 1 cm lengths, and then mixed back with the soil. So, the inocula from *C. korshinskii* (A), *H. leave* (B) and *C. microphylla* (C) consisted of AM-colonized root pieces, spores, and hyphae originated. The number of spores in the inocula A, B and C were 382 ± 57, 325 ± 33, and 367 ± 76 AMF spores per 100 g inoculum respectively (Figure S1). No significant differences in the spore density were detected among them. Though AM fungal communities could change during the trap culture, the influence was minor considering the relative short-term conditioning time [38].

### 2.3. Seedling Preparation

Seeds of *C. korshinskii* and *C. microphylla* were collected at the study site at the same time as soil sampling. In 2016, 1000 plump seeds were disinfected with 75% alcohol, washed with sterilized tap water and then pre-germinated in 100 pots (i.e., 10 seeds per pot) for each shrub using the same sterilized growth substrate as in the AMF trap culture. After germination, the number of seedlings in each pot was thinned to 3 and were cultivated in a greenhouse for 2 months from May to June to make sure that seedlings were strong enough to survive after transplanting to the field.

### 2.4. Field Experiment Design

In the July of 2016, an area of 8 m × 15 m was fenced in the study site to setup the field experiment. Plants grown in the fenced area were removed manually, and the field was tilled to potentially disrupt the hyphae of AMF and create a uniform environment. Then the fenced area was separated to 112 subplots (0.5 m length × 0.5 m width × 0.1 m depth) with 0.5 m distance from each other.

The seedlings with a good status and nearly equal height (about 15 cm) obtained from seedling preparation were transplanted to field site. Each subplot received 3 *C. korshinskii* or *C. microphylla* seedlings randomly. AMF inocula from the trap culture were applied at the time of seedling transplantation. Briefly, a small hole was dug by sterilized spade, and 100 g of inoculum (average 358 AMF spores, Figure S1) were put on the bottom and then seedlings were placed on the top of the inoculum. Weeds in the field were controlled manually twice a year in June and September. Thus, the field experiment consisted of 2 factors including 2 host species and 4 AMF inocula with a replicate of 14. There were 112 microcosms in total in this study (i.e., 2 host species × 4 AMF inocula × 14 replicates). The 4 AMF treatments included inocula originated from *C. korshinskii* (A), *H. leave* (B) and *C. microphylla* (C), adding sterilized-inocula (mixture of A, B, and C) as control (CK).

### 2.5. Field Measurements

In July 2017 and 2018, the height and canopy diameter of plants were measured by steel tape. Gas-exchange characteristics, including photosynthetic rate, transpiration rate, and stomatal conductance (GH_2_O) were measured on leaves in situ with a portable leaf gas exchange system (GFS-3000, Walz GmbH, Effeltrich, Bavaria, Germany). All gas-exchange characteristics were measured between 9:00 and 11:30 (24 h) on sunny days and data were measured at a temperature between 25–30 °C, relative humidity about 70%, and CO_2_ concentration inside the chamber of ambient level approximate 400 ppm.

In July 2017, soil cores (5 cm diameter) were collected to a depth of 20 cm in the root area of the plants. For each treatment, 10 of 14 replicates with nearly equal plant height were sampled adding up to a total of 80 samples. For each sample, the plant roots were collected and transferred to the lab with ice. After gentle washing of the roots with tap water, a subset of the roots was stained with trypan blue and checked for AMF colonization rate using the magnified gridline intersect method [39]. An additional subset of roots was stored at −20 °C for subsequent molecular analysis.

Plants were harvested in August 2019. Plant height was measured before harvest and dry (65 °C, 72 h) weights of shoots were recorded to determine the average shoot biomass per individual. Dried leaf and shoot tissues were ground to powder before analyzing for total N and P. Foliar P was determined colorimetrically after digestion with sulfuric acid using the molybdenum blue method [40] and foliar N was determined by the Kjeldahl method [41].

### 2.6. DNA Extraction and Sequencing

Total DNA of the root samples was extracted by the CTAB (cetyltrimethylammonium bromide) protocol [42]. Briefly, roots were cut into 1 cm pieces and 20 fragments of them were randomly picked up for DNA extraction. We also extracted DNA from 0.25 g fresh soil samples of three AMF inocula after trap culture. 5 replicates of each inoculum were extracted using DNA extraction kits (DNeasy PowerSoil, Qiagen, Valencia, CA, USA) according to the manufacturer’s protocol.

Glomeromycotina sequences were amplified by nested PCR with the SSU rRNA gene primers NS31-AML2 and AMV4.5NF–AMDGR as previous study [43]. We used the Quantitative Insights into Microbial Ecology (QIIME v1.7.0) [44] and the UPARSE pipeline [45] to treat raw sequences. In brief, sequences below the quality score of 20 and fewer than 200 bp in length were excluded. Primer-free sequences were de-replicated and chimeric sequences were removed. The sequences were binned into operational taxonomic units (OTUs) with 97% similarity in USEARCH version 11 [46], and the most abundant sequence from each OTU was selected as a representative sequence for that OTU. Then, a Basic Local Alignment Search Tool (BLAST) search against the GenBank database at the National Center for Biotechnology Information (NCBI) to detect non Glomeromycotina sequences. Rare OTUs with relative abundance less than 0.01% (total abundance less than 510) were also removed, resulting 100 abundant AMF OTUs for downstream analysis. Representative sequences of each OTU in this study were deposited in GenBank (MK318664-MK318763). Further, the BLAST function was used to retrieve closely related reference Glomeromycotina sequences for the representative sequences of OTUs. Neighbor-joining (NJ) phylogenetic analysis [47] was computed in MEGA version 7 with 1000 bootstrap replicates to evaluate the tree and with *Henningsomyces candidus* as outgroup to root the tree.

### 2.7. Statistic Analysis

Statistical analysis was conducted using R (version 3.5.0). Analysis of variance (ANOVA) was performed to test for differences in plant responses to each inoculation treatment of the two Caragana plants, followed by Tukey’s HSD post-hoc analysis. Within the one-way ANOVA, multiple comparisons were done with orthogonal, a priori contrasts (inoculated vs. un-inoculated and home vs. away) to test if there was a net inoculation effect overall and if there was a home advantage under home AMF and plant combinations.

AM fungal community’s Shannon–Wiener diversity and species richness were calculated using the vegan package [48]. Indicator species analysis of AMF was performed using indicspecies [49]. Permutational multivariate analysis (PERMANOVA) was used to test whether host species (*C. korshinskii* and *C. microphylla*) and inoculum sources (A, B, C, and CK) explained differences in AM fungal community (sequence number dataset, wisconsin-square root transformed), and we visualized these differences at a community level using nonmetric multidimensional scaling (NMDS) using Bray–Curtis distances. Linear regression was performed to explore relationships between plant variables and AMF abundance. Additionally, to estimate the causal effects of AMF inoculation on host plant performance, structural equation models (SEM) were calculated using SPSS Amos v. 23.0.

## 3. Result

### 3.1. AM Fungal Community Properties

**Figure 1:**
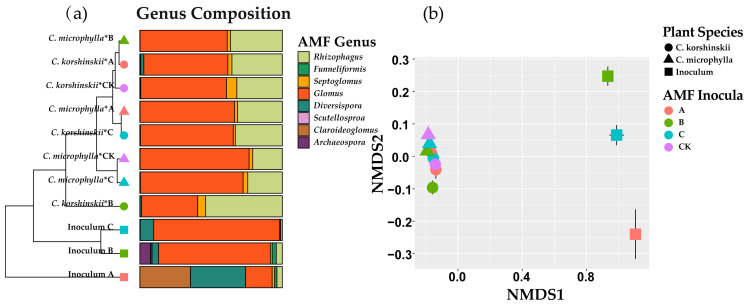
Relative abundance of AM fungal genus in original inocula (A: *C. korshinskii*, B: *H. leave*, C: *C. microphylla*, and CK: sterilized inocula) and plant roots after one year of growth (**a**), and non-metric multidimensional scaling (NMDS) ordination plot illustrating differences in AMF OTU composition among the original inocula and plant roots (**b**).

All samples of inocula yielded positive PCR products of the expected size (250 bp) and the rarefaction curve suggested that our sampling captured most of the community (Figure S2). Differences in Shannon diversity (F = 3.848, *p* = 0.058) and composition of AMF community (R^2^ = 0.400, *p* = 0.075) were detected among the three inocula. Inoculum B showed the highest AMF diversity relative to inoculum A and C (Figure S3). 100 OTUs could be assigned to putative AMF and all the identified OTUs were affiliated with genus levels within the families Archaeosporaceae, Claroideoglomeraceae, Diversisporaceae, Gigasporaceae, and Glomeraceae. *Diversispora* was significantly enriched in inoculum A (Figure S4), but *Archaeospora* was significantly enriched in inoculum B. *Glomus* was significantly more abundant in inoculum C (Table S1). The NMDS analysis also showed the dramatically different in AM fungal community among the three inocula (Figure 1).

In addition, 92 of 100 AMF OTUs were found associated with *Caragana* roots. Though AMF richness was neither influenced by host plant species nor inoculum sources, the Shannon diversity was significantly influenced by inoculum sources (F = 5.027, *p* = 0.003) and their interaction (F = 3.901, *p* = 0.012) (Figure S5). The community structure of AMF associated with *C. korshinskii* and *C. microphylla* roots was significantly influenced by both inoculum sources (F = 1.558, *p* = 0.033) and plant species (F = 8.528, *p* = 0.001) according to PERMANOVA result. AM fungal communities inside *C. korshinskii* and *C. microphylla* roots were significantly different.

### 3.2. Plant Response to AMF Inoculation

**Figure 2:**
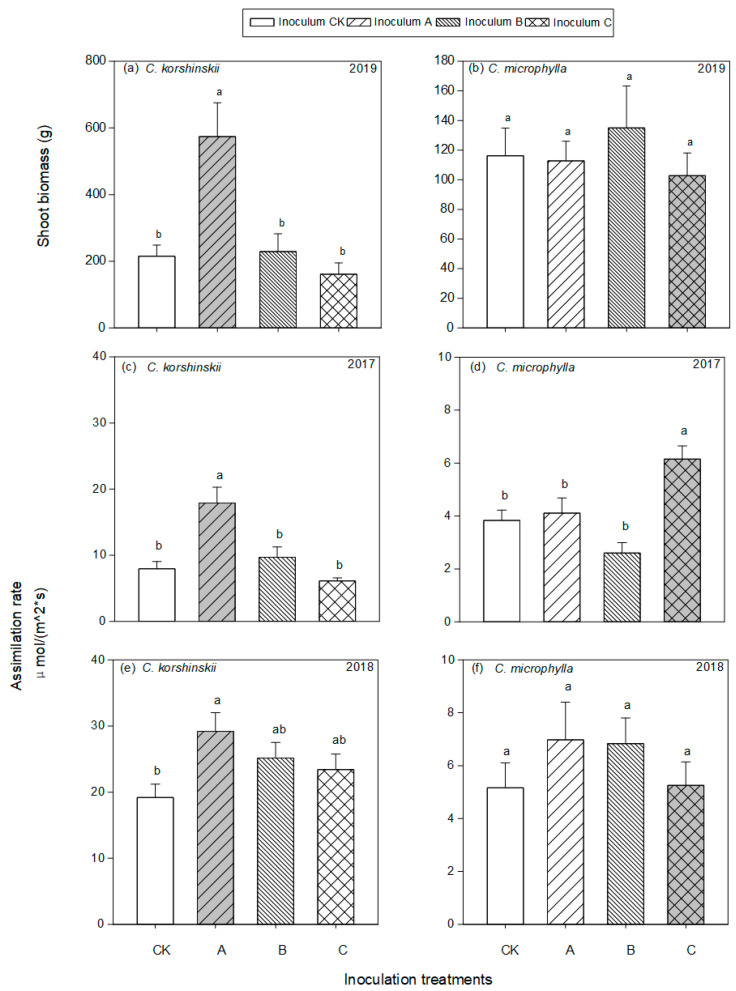
Shoot biomass (**a**,**b**) and photosynthetic rate (**c**–**f**) of *C. korshinskii* and *C. microphylla* plants in 3 years. Inoculation treatments including inocula originating from rhizosphere of *C. korshinskii* (A), *H. leave* (B), *C. microphylla* (C) and sterilized control (CK). Bars indicate means with standard error (*n* = 7 for shoot biomass and *n* = 14 for photosynthetic rate). Different letters above bars indicated significant differences at *p* < 0.05 level according to Tukey’s test. Grey bars indicated home AMF and plant combinations.

Host species, AMF inoculation, and experimental year showed significant effects on most of the plant measurements (Table 1). *C. korshinskii* was significantly higher in shoot biomass (F = 25.276, *p* < 0.001) relative to *C. microphylla*, while foliar N (F = 53.833, *p* < 0.001) and P (F = 9.538, *p* = 0.003) showed reverse pattern (Table S2). AMF inoculation significantly increased the shoot biomass and photosynthetic rate of *C. korshinskii* by 49.53%, and 26.96% relative to CK, respectively. However, no significant effects of AMF inoculation were found for *C. microphylla*, except for increased photosynthetic rate by 11.74% in 2017 (Figure 2).

Furthermore, the different responses of *C. korshinskii* to home and away AMF inoculation was found (Table 2). Home AMF significantly increased the shoot biomass, foliar N, and foliar P of *C. korshinskii* by 194.35%, 15.20%, and 25.69% relative to away AMF inoculation, respectively (*p* < 0.001, *p* = 0.002, *p* = 0.009). However, no significant differences in *C. microphylla* responses to home and away AMF inoculation, except for increased photosynthetic rate in 2017 (*p* < 0.001) and Foliar N:P (*p* = 0.003).

### 3.3. The Association between AM Fungal Community and Plant Performance

**Figure 3:**
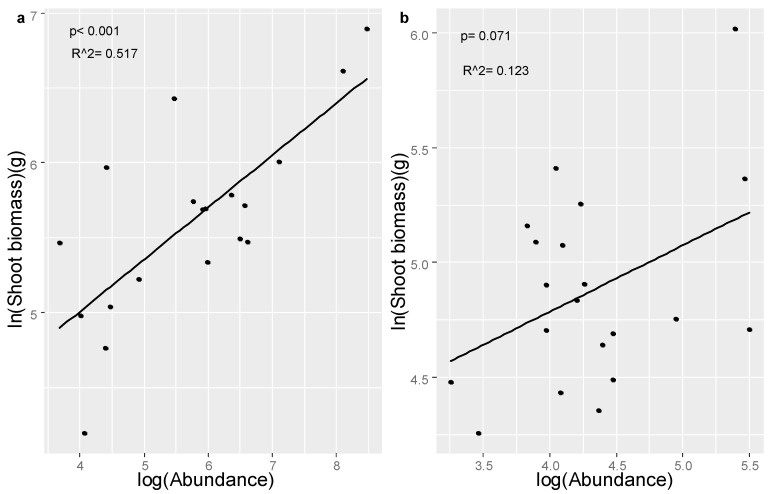
Correlations between shoot biomass of *C. korshinskii* ((**a**), *n* = 19) and *C. microphylla* ((**b**), *n* = 20) and the corresponding relative abundance of AMF *Diversispora*. Both plant biomass and AMF abundance were log transformed. Statistically significant effects (*p*) and the coefficient of determination (R^2^) were presented.

Linear regression revealed a significant positive relationship between *C. korshinskii* shoot biomass and the Shannon diversity of the AMF (R^2^ = 0.282, *p* = 0.019, Figure S6) and between *C. microphylla* shoot biomass and the richness of associated AM fungal communities (R^2^ = 0.219, *p* = 0.037, Figure S7). Furthermore, significant positively relationship between *C. korshinskii* shoot biomass and the abundance of AMF genus *Diversispora* was detected (R^2^ = 0.517, *p* < 0.001, Figure 3). The abundance of *Diversispora* was also marginally correlated with the shoot biomass of *C. microphylla* plants (R^2^ = 0.123, *p* = 0.071).

**Figure 4:**
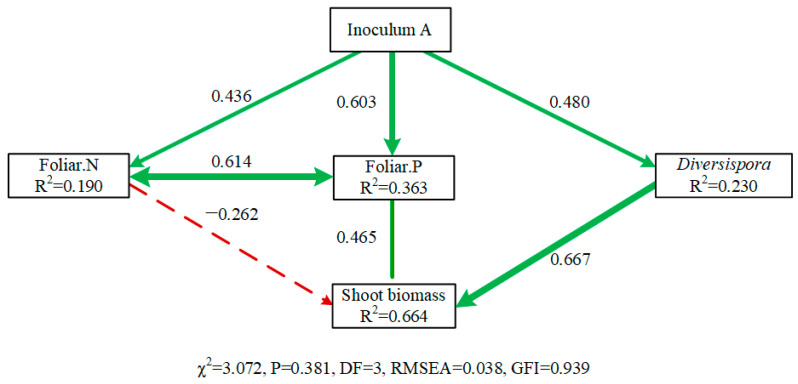
Structural equation model (SEM) showed home AMF inoculation effects on *C. korshinskii* plant performance. Solid and dashed lines indicated significant (*p* < 0.05), and non-significant pathways (*p* > 0.05). The double headed arrow indicated correlations. Numbers above the arrows showed the standardized path coefficients. Arrow width is proportional to the strength of different pathways. Green and red lines indicated positive and negative pathways. R^2^ below each dependent variable represented the proportion of variance explained.

The SEM results with a good fit showed the effects of home AMF inoculation on plant performance for *C. korshinskii* plants (Figure 4). The models explained 66.4% of the variance in plant shoot biomass and the path coefficients (λ) for direct effects were displayed (Table S3). According to the standardized total effects, the abundance of *Diversispora* AMF (λ = 0.667) was the strongest predictor for plant shoot biomass. The effect of home AMF inoculation (inoculum A) on plant shoot biomass was mainly through the indirect path mediated by the influence of P uptake, and the abundance of AMF genus *Diversispora*.

## 4. Discussion

Beneficial effects of conspecific (home) AMF communities on shrub growth were detected here. These results suggested that native AMF might aid revegetation efforts in the desertified grasslands in north China. However, the different host origin effects of native AM fungal communities on plant growth and nutrition revealed that plant performance was depending on both host plant identity and fungal partners, and home advantage for conspecific AM fungal community was proved only for *C. korshinskii* plants.

Our first hypothesis was that native AMF inoculation would enhance plant performance. In this study, *C. korshinskii* plants showed increased shoot biomass when inoculated with native AM fungal communities, which was in line with previous report [50]. However, the effect of AMF inoculation also depended heavily on the host plant identity as has been reported [51]. The inoculation only increased the biomass of *C. korshinskii* plants, but not for *C. microphylla*. The fact that un-inoculated *C. microphylla* plants had higher nutrient also showed that the feedback between plant and AMF was host specific.

The different effects of home AMF on *C. korshinskii* and *C. microphylla* might be due to the differences in their physiology and interaction specificity to AMF [36]. Naturally in the Mongolia plateau, *C. korshinskii* and *C. microphylla* form a geographical cline with *C. korshinskii* distributed in extremely dry regions where soil water content is low and temperature is high. Whereas *C. microphylla* plants are distributed in semi-arid regions with relatively higher humidity [52]. Further studies on the physiological characteristics of these species revealed that *C. korshinskii* were able to maintain high photosynthetic rate and water use efficiency in more stressed environment compared to *C. microphylla* plants [53,54,55]. Thus, the *C. korshinskii*-AMF symbioses might be important for adaption of *C. korshinskii* to desert condition. And in this study, the higher photosynthetic rate of *C. korshinskii* species implied its higher ability to allocate more carbon to its fungi partner than that of *C. microphylla* plants. This may explain the higher colonization rate and more sensitive response of *C. korshinskii* species to AMF inoculation relative to *C. microphylla*. Regardless of why the two legume species showed varied response to AMF inocula, the inoculation effect holds implications for revegetation that the introduction of beneficial microorganism such as native AMF should be considered.

For the second hypothesis, the three AMF inocula had different effects on plant performance and the functional differences of native AM fungal communities from the three host rhizospheres were proved. Inoculum A produced the highest photosynthetic rate and the highest shoot biomass for *C. korshinskii* when compared with other inocula. Plants are known to have varied response to either different species of AMF, or different AM fungal communities [56,57,58]. For instance, AMF collected from abandoned field or grassland had varied effects on the plants [59]. Likewise, another study found that AMF collected from the field with different levels of long-term manure addition differently affect plant shoot biomass and P concentration [60]. Specifically, the situation when native AMF enhanced the plant growth more than the foreign one was described as “local adaptation” [16], and lots of studies reported that AMF locally adapted to the environment would be more beneficial [61,62,63,64]. Our results showed that the home AMF treatment (i.e., inoculum A for *C. korshinskii*) increased the biomass, N and P content of their plant host, indicating home advantage.

To further explore the home advantage of *C. korshinskii* and the corresponding AMF, molecular analysis was performed. Overall, we detected 8 genera among inocula and *Caragana* plant roots. Indicator species analysis showed that the genus *Diversispora* was the indicator of inoculum A and its abundance was significantly higher than the other two inocula. Interestingly, the abundance of *Diversispora* retrieved from the colonized roots was positively correlated with the plant biomass at harvest. Taken together, *Diversispora* that came from the inoculum may contribute to the biomass promotion, in line with previous findings [65]. It has been known that AMF differed in allocating biomass to intraradical hyphae, extraradical hyphae and spores [66,67]. Recent study found that edaphophilic AMF such as *Diversispora* with high investment to extraradical hyphae were more abundant in shrub roots [68]. Though we could not assure that *Diversispora* directly from the inoculum colonized *Caragana* roots well, according to the SEM, the home AMF treatment (inoculum A) had a positive effect on the abundance of *Diversispora* and the *Diversispora* also had a positive effect on plant biomass. Taken together, *Diversispora* might be the key AMF group in *C. korshinskii* growth in desertified areas. Previous study inoculating plants with AMF from *Diversispora* showed the highest biomass compared with other two species from *Paraglomus* and *Claroideoglomus* [69], but knowledge regarding the systemic functional difference among AMF genera affiliated with different guilds were still insufficient. Further study should further explore the different roles of edaphophilic and rhizophilic AMF to optimize the inoculation benefits.

## 5. Conclusions

In conclusion, this study explored the relationship between *Caragana* plant performance and the home advantage of conspecific AM fungal community in the Kubuqi Desert. *C. korshinskii* tend to show better performance when inoculated with home AMF, which may partly relate to *Diversispora*. We demonstrated that the growth of leguminous *C. korshinskii* can be enhanced in the desert with the help of conspecific native AMF community. Considering the vital roles AMF played, the reconstitution of the soil fungi may be a key step in restoration programs for such desert environments. For further studies, large scale field experiments containing different combinations of AMF community and host plants are needed and the evaluation on long-term effects of the introduced AMF on plants and soil should also be considered.

## Figures and Tables

**Table 1:** Results of three-way analysis of variances showing the effects of host plant species, AMF inoculation, year and their interactions on plant performance.

	Height	Canopy	Photosynthetic Rate	Transpiration Rate	Vapor Pressure Deficit	Stomatal Conductance	Colonization Rate	Shoot Biomass	Foliar N	Foliar P	Foliar N:P
Host	<0.001	<0.001	<0.001	<0.001	<0.001	<0.001	0.2394	<0.001	<0.001	0.003	0.011
Inoculation	<0.001	0.001	0.001	0.189	0.000	0.002	0.0241	<0.001	0.102	0.163	0.076
Year	<0.001	<0.001	<0.001	<0.001	<0.001	<0.001					
Host:Inoculation	<0.001	0.001	0.029	0.026	0.501	0.015	0.0646	0.0119	0.0406	0.0130	0.5085
Host:Year	<0.001	0.012	<0.001	<0.001	<0.001	0.002					
Inoculation:Year	0.026	0.001	0.584	0.435	0.307	0.213					
Host:Inoculation:Year	0.769	0.341	0.035	0.151	0.033	0.109					

**Table 2:** Results of orthogonal contrasts for photosynthetic rate, biomass, and nutrient content of Caragana species among inoculated or un-inoculated and inoculated with home AMF (home) or other inocula (away). ‘+/−’ means a positive/negative effect of AMF inoculation or home advantage of conspecific inocula. Statistically significant effects (P) were shown behind.

	*C. korshinskii*				*C. microphylla*			
	Inoculated vs. Un-Inoculated		Home vs. Away		Inoculated vs. Un-Inoculated		Home vs. Away	
Shoot biomass	+	0.144	+	<0.001	+	0.977	−	0.38
Photosynthetic rate (2017)	+	0.132	+	<0.001	+	0.376	+	<0.001
Photosynthetic rate (2018)	+	0.017	+	0.102	−	0.426	−	0.144
Foliar N	+	0.854	+	0.002	−	0.002	+	0.089
Foliar P	+	0.244	+	0.009	−	0.008	−	0.444
Foliar N:P	−	0.397	−	0.365	+	0.383	+	0.003

## Data Availability

Available upon reasonable request.

## Supplementary Materials

The following are available online at https://www.mdpi.com/article/10.3390/jof7121077/s1, Figure S1. Spore density of AMF in soil samples taken in 2014 (a) and AMF inoculum A, B and C prepared in 2016 (b). Spores were extracted from 20 g soil samples and then counted. Figure S2. Rarefaction curves of AMF species richness along the number of sequences obtained from the 3 inocula (a) and rhizospheres of 2 *Caragana* plant roots (b). The rarefaction curve suggested that our sampling captured most of the AMF community. Figure S3. S Shannon diversity (a) and species richness (b) of AMF community associated with inoculum A, B, and C. No significant difference was found among the inoculum A, B and C. Figure S4. The relative abundance of AMF belonging to *Diversispora* associated with 3 inocula. Apparently, *Diversispora* was significantly enriched in inoculum A. Figure S5. Shannon diversity of AMF associated with *C. korshinskii,* and *C. microphylla* plants with different AMF inoculation treatments. Inoculation treatments including inocula originating from rhizosphere of *C. korshinskii* (A), *H. leave* (B), *C. microphylla* (C), and sterilized control (CK). Figure S6. Linear regression indicating a significant positive relationship between *C. korshinskii* plant shoot biomass and the Shannon diversity of rhizosphere AMF community. Figure S7. Linear regression indicating a significant positive relationship between *C. microphylla* plant shoot biomass and the richness of rhizosphere AMF community. Table S1. Results of indicator species analysis of AMF genus associated with 3 inocula. Table S2. Foliar and shoot nutrient of Caragana species under different AMF treatments. Table S3. Standardized total effects of the SEM.
