# Supplementary material for: Host-Specific Effects of Arbuscular Mycorrhizal Fungi on Two Caragana Species in Desert Grassland
Source: J Fungi (Basel). 2021 Dec 15;7(12):1077. doi: 10.3390/jof7121077 (PMC8708327; doi:10.3390/jof7121077)
Supplement: Supplementary file 1 [file jof-07-01077-s001.zip › jof-1480709-supplementary.pdf]

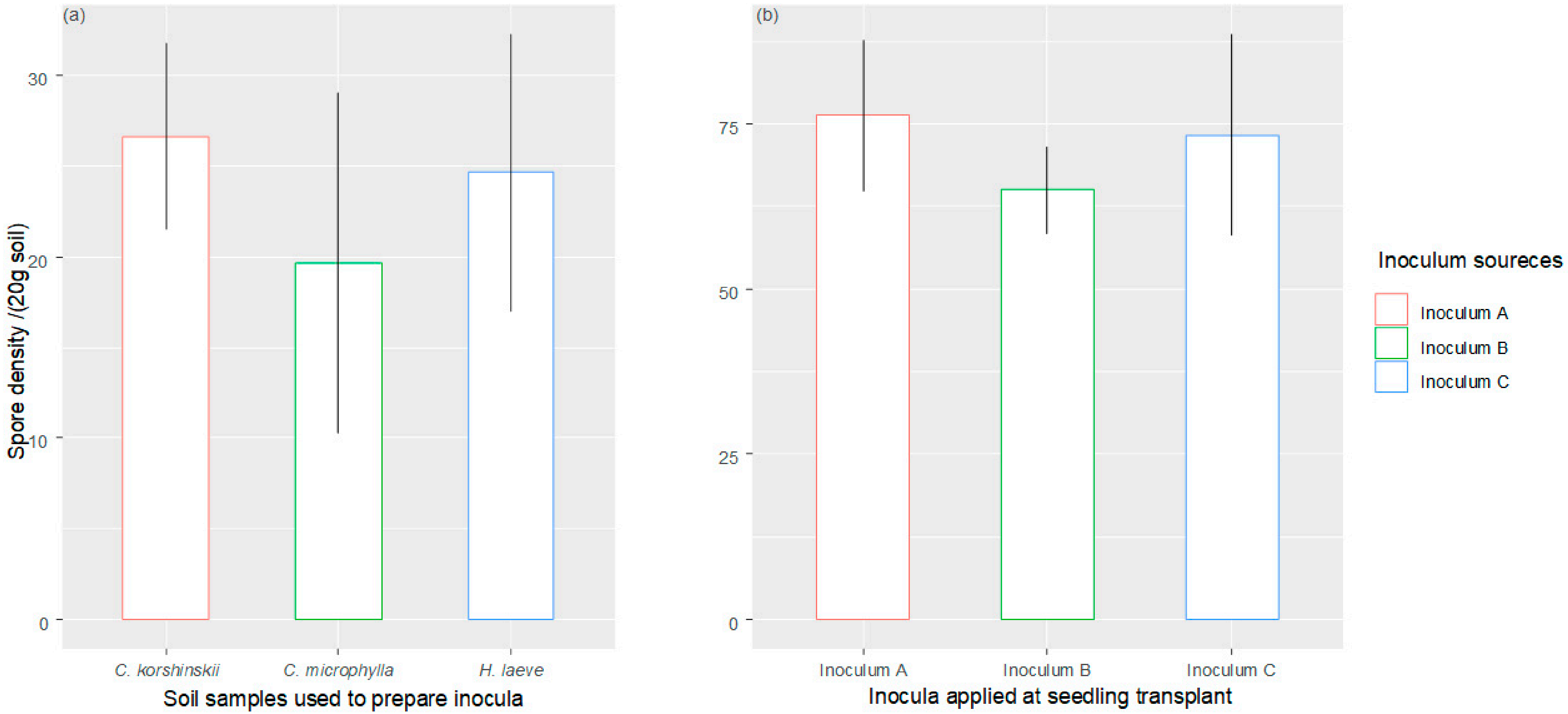

Figure S1. Spore density of AMF in soil samples taken in 2014 (a) and AMF inoculum *C. korshinskii* (A), *H. leave* (B) and *C. microphylla* (C) prepared in 2016 (b). Spores were extracted from 20g soil samples and then counted.

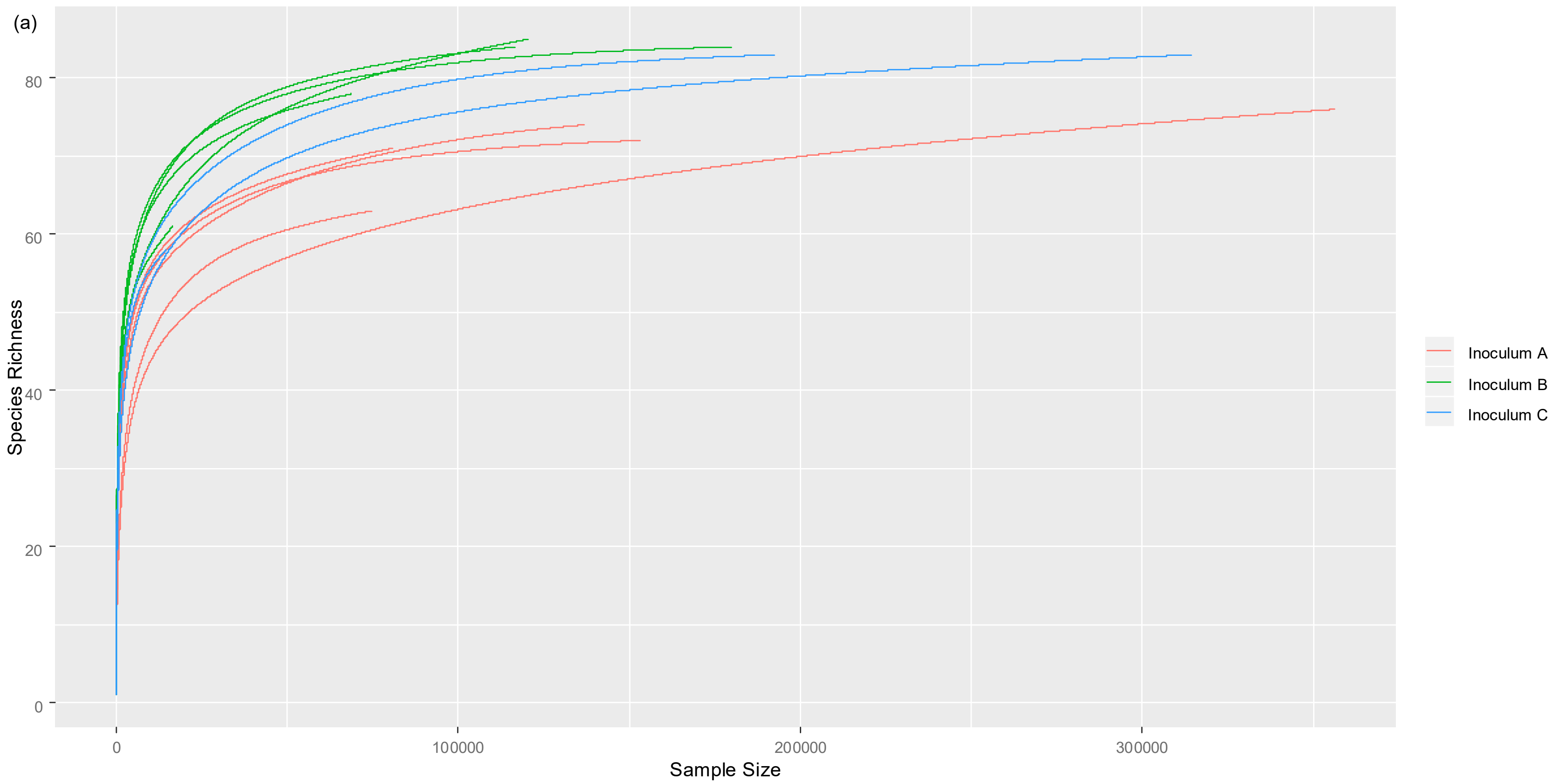
(a)
Species Richness
0
20
40
60
80
0
100000
200000
300000
Sample Size
Inoculum A
Inoculum B
Inoculum C

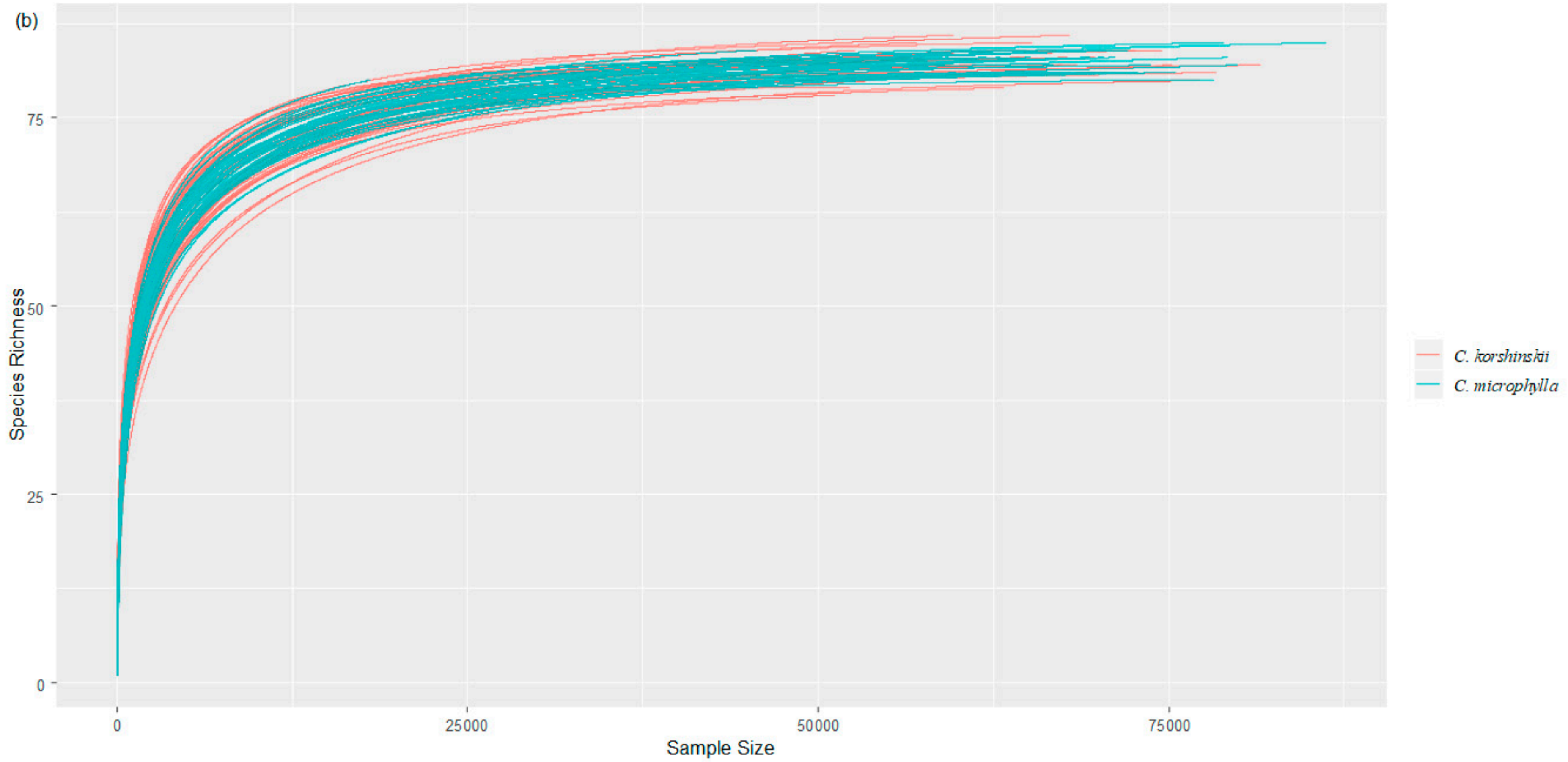

Figure S2. Rarefaction curves of AMF species richness along the number of sequences obtained from the 3 inocula (a) and rhizospheres of 2 *Caragana* plant roots (b). The rarefaction curve suggested that our sampling captured most of the AMF community.

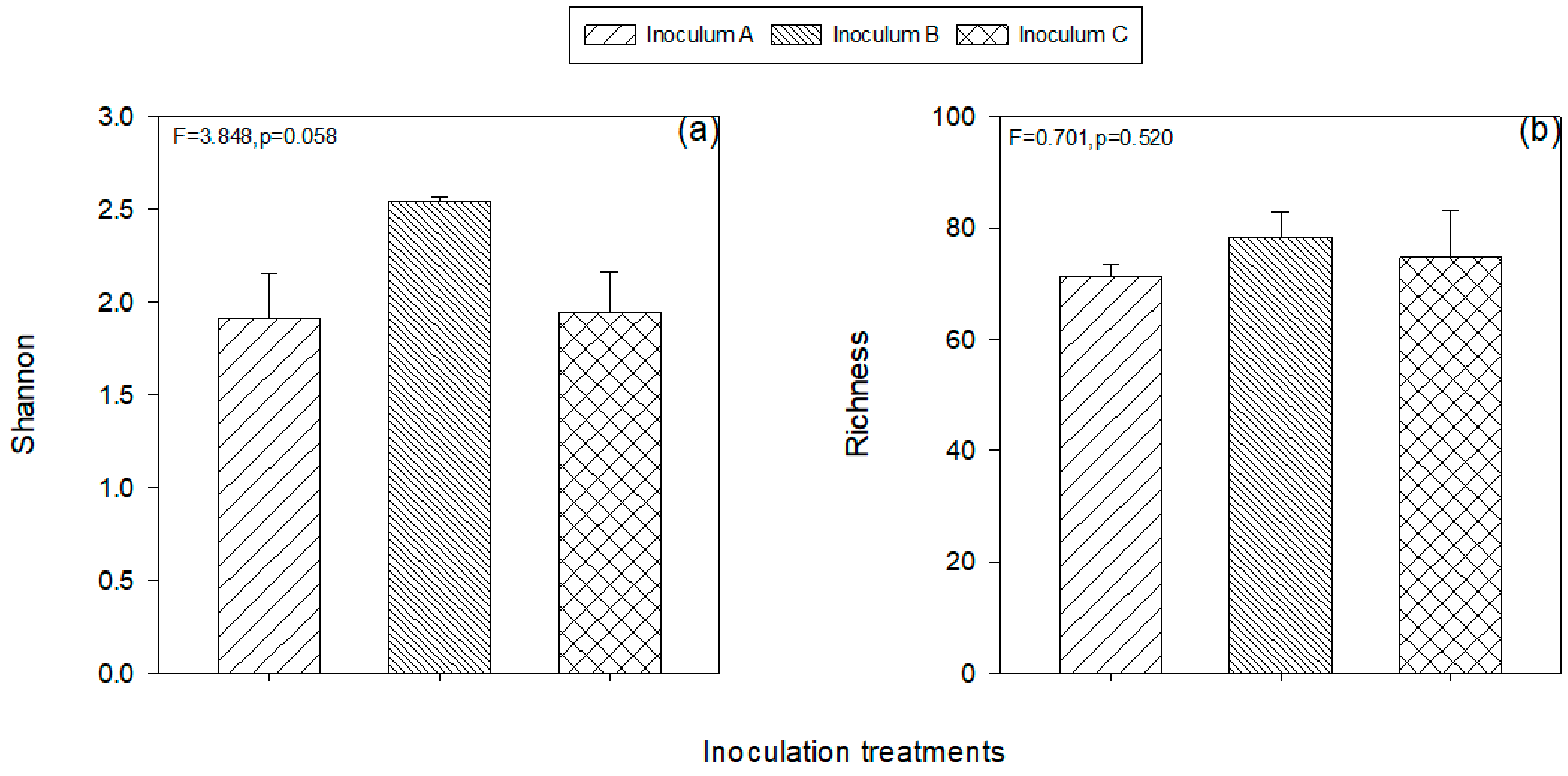

Figure S3. Shannon diversity (a) and species richness (b) of AMF community associated with inoculum A, B, and C. No significant difference was found among the inoculum A, B and C.

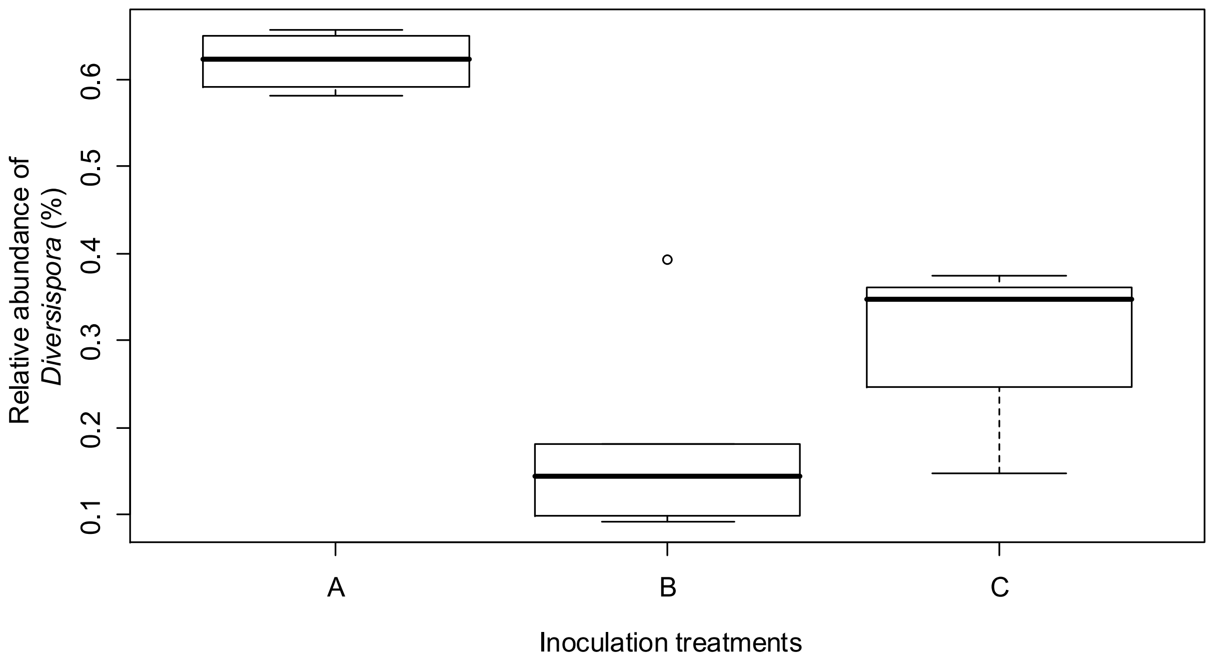

Figure S4. The relative abundance of AMF belonging to *Diversispora* associated with 3 inocula. Apparently, *Diversispora* was significantly enriched in inoculum A.

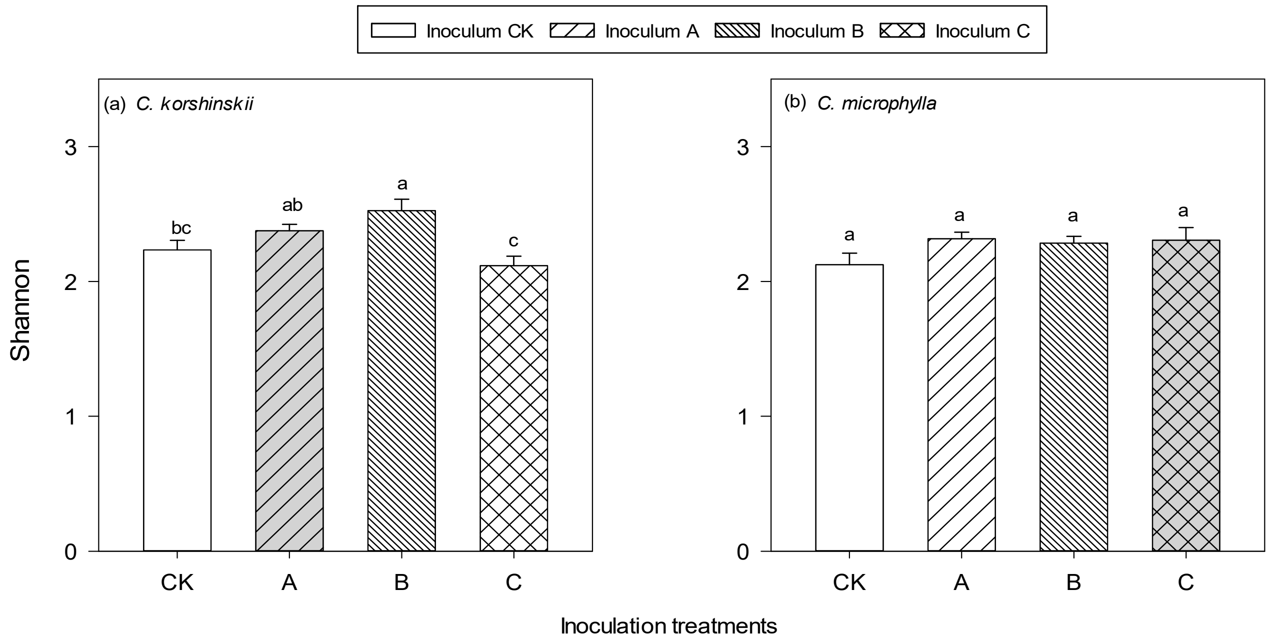

Figure S5. Shannon diversity of AMF associated with *C. korshinskii,* and *C. microphylla* plants with different AMF inoculation treatments. Inoculation treatments including inocula originating from rhizosphere of *C. korshinskii* (A), *H. leave* (B), *C. microphylla* (C), and sterilized control (CK). Bars indicate means with standard error(n=10). Different letters above bars indicated significant differences at P< 0.05 level according to Tukey's test. Gray bars indicated home AMF and plant combinations. A two-way ANOVA revealed significant effects of inoculum sources (F=5.027, P=0.003) but not host plant species on the Shannon diversity of AMF. The interaction between host plant species and inoculum sources was not significant (F=3.901, P=0.012).

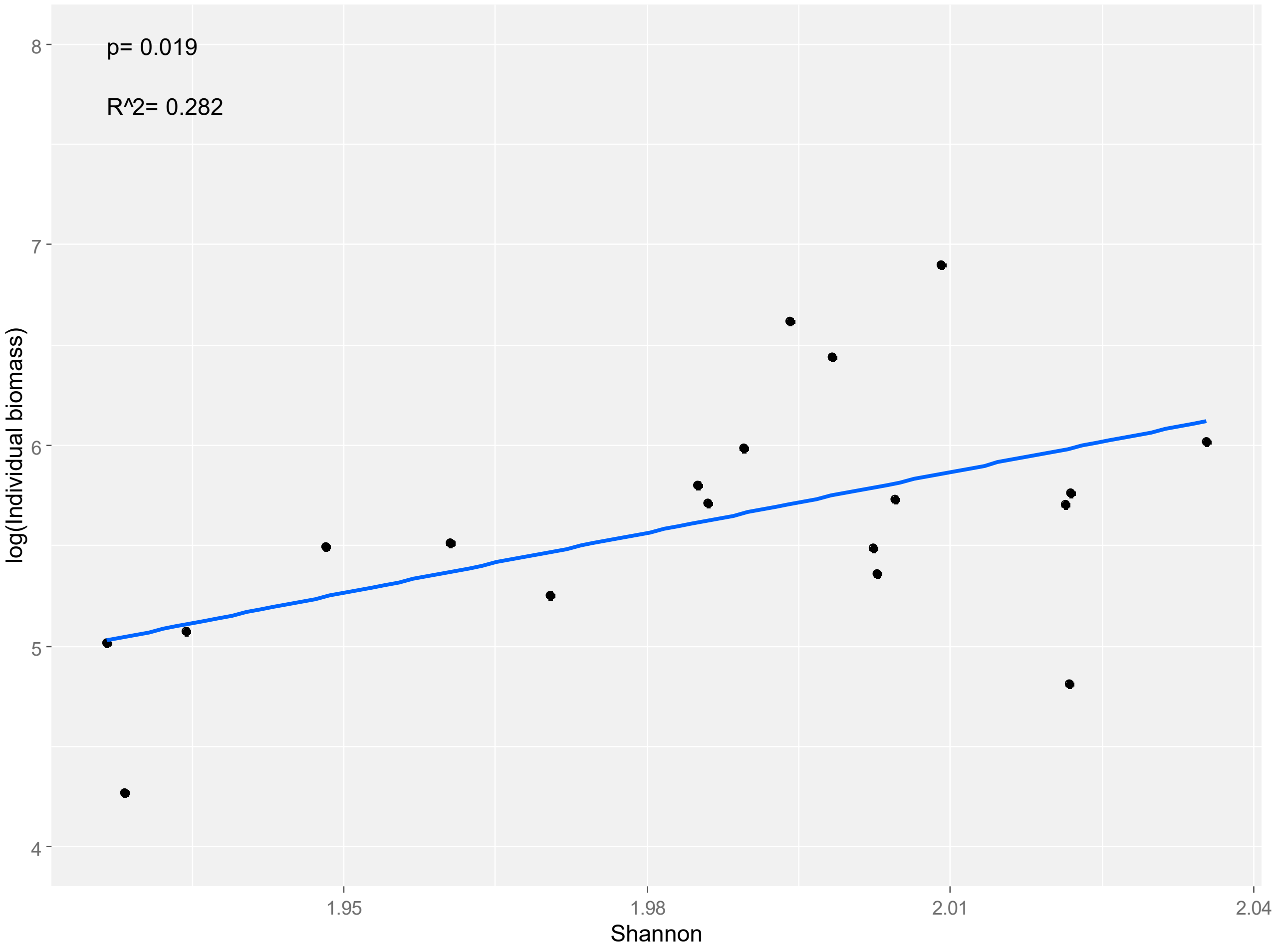

Figure S6. Linear regression indicating a significant positive relationship between *C. korshinskii* plant shoot biomass and the Shannon diversity of rhizosphere AMF community.

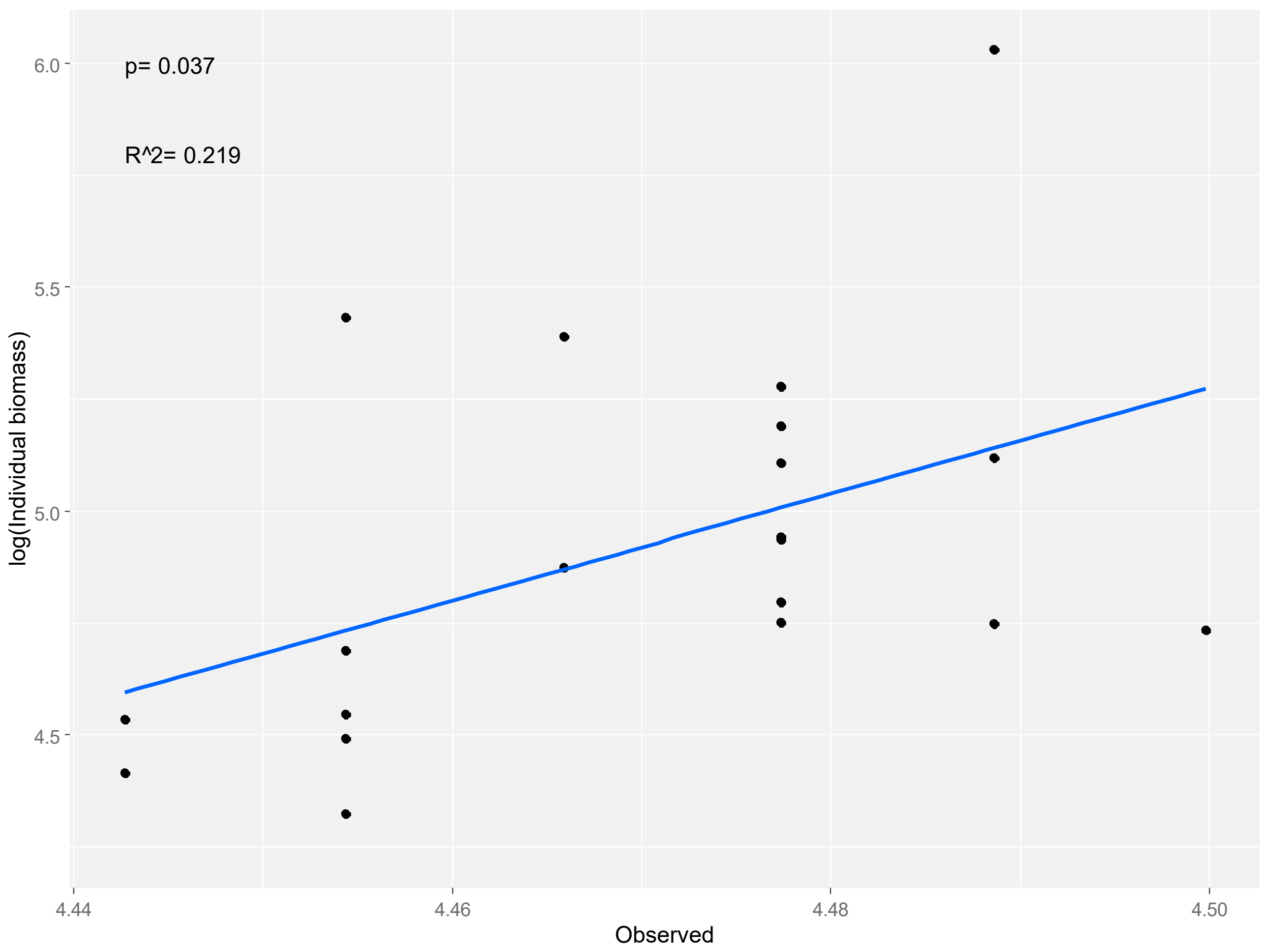

Figure S7. Linear regression indicating a significant positive relationship between *C. microphylla* plant shoot biomass and the richness of rhizosphere AMF community.

Table S1. Results of indicator species analysis of AMF genus associated with 3 inocula. Strongly correlated with the plant biomass, *Diversispora* was the indicator of inoculum A.

Genus	A	B	C	best	P
Glomus	0.9999	0.1245	0.0081	C	0.0241
Diversispora	0.0004	0.9866	0.8453	A	0.0012
Claroideoglomus	0.0003	0.9566	0.8882	A	0.0009
Archaeospora	0.9562	0.0001	0.9981	B	0.0003

Table S2. Results of indicator species analysis of AMF OTUs associated with two host species. Among 35 significant indicators OTUs, 17 OTUs were the indicator of *C. korshinskii* and 18 OTUs were the indicator of *C. microphylla.*

	C. korshinskii	*C. microphylla*	best	P
OTU_122	1.0000	0.0001	2	0.0002
OTU_10	1.0000	0.0001	2	0.0002
OTU_117	1.0000	0.0001	2	0.0002
OTU_101	1.0000	0.0002	2	0.0004
OTU_27	1.0000	0.0001	2	0.0002
OTU_23	0.9762	0.0239	2	0.0472
OTU_70	0.0003	0.9998	1	0.0006
OTU_3	0.0001	1.0000	1	0.0002
OTU_15	0.0005	0.9996	1	0.0010
OTU_174	0.0011	0.9990	1	0.0022
OTU_100	0.0016	0.9985	1	0.0032
OTU_107	0.0002	0.9999	1	0.0004
OTU_17	0.0002	0.9999	1	0.0004
OTU_45	0.0131	0.9875	1	0.0260
OTU_139	0.0241	0.9776	1	0.0476
OTU_181	0.9999	0.0002	2	0.0004
OTU_55	0.9999	0.0003	2	0.0006
OTU_124	0.9954	0.0047	2	0.0094
OTU_151	1.0000	0.0001	2	0.0002
OTU_190	1.0000	0.0001	2	0.0002

OTU_83	0.9983	0.0019	2	0.0038
OTU_143	1.0000	0.0001	2	0.0002
OTU_1	0.9990	0.0011	2	0.0022
OTU_183	0.9985	0.0017	2	0.0034
OTU_104	1.0000	0.0001	2	0.0002
OTU_148	1.0000	0.0001	2	0.0002
OTU_172	1.0000	0.0001	2	0.0002
OTU_42	0.0001	1.0000	1	0.0002
OTU_24	0.0001	1.0000	1	0.0002
OTU_73	0.0001	1.0000	1	0.0002
OTU_114	0.0001	1.0000	1	0.0002
OTU_16	0.0001	1.0000	1	0.0002
OTU_208	0.0178	0.9948	1	0.0353
OTU_6	0.0055	0.9979	1	0.0110
OTU_19	0.0156	0.9861	1	0.0310

Table S3. Standardized total effects of the SEM. According to the total effects, inoculum A influenced the *C. korshinskii* plant N, P untake and shoot biomass.

	Inoculum A	*Diversispora*	Foliar.P	Foliar.N
Diversispora	0.480	0.000	0.000	0.000
Foliar.P	0.603	0.000	0.000	0.000
Foliar.N	0.436	0.000	0.000	0.000
Shoot biomass	0.486	0.667	0.465	-0.262
